# Cardiovascular exercise enhances motor learning across multiple sessions in people with Parkinson’s disease: a randomized controlled pilot trial

**DOI:** 10.1038/s41539-025-00391-6

**Published:** 2025-12-23

**Authors:** Philipp Wanner, Nicole Frisch, Samuel Rikus, Marc Roig, Simon Steib

**Affiliations:** 1 Human Movement, Training and Active Aging Department, Institute of Sports and Sports Sciences, Heidelberg University, Heidelberg, Germany; 2 Memory and Motor Rehabilitation Laboratory (MEMORY-LAB), Feil and Oberfeld Research Centre, Jewish Rehabilitation Hospital, Montreal Centre for Interdisciplinary Research in Rehabilitation (CRIR), Laval, QC Canada; 3 School of Physical and Occupational Therapy, McGill University, Montréal, QC Canada; 4 Network Aging Research, Heidelberg University, Heidelberg, Germany

**Keywords:** Motor control, Basal ganglia, Cognitive ageing, Learning and memory, Consolidation, Long-term memory, Neural ageing

## Abstract

Motor learning is critical for effective motor rehabilitation, yet impaired in people with Parkinson’s Disease (pwPD). Emerging evidence suggests that cardiovascular exercise (CVE), performed close to skill practice, may promote brain plasticity and motor learning. However, research has predominantly focused on acute effects of a single CVE session in neurotypical individuals. Here, we examined whether post-practice CVE enhances motor learning over multiple weeks. Twenty-four pwPD were randomly assigned to either moderate-intensity cycling or seated rest after practicing a novel balance task across six sessions. As hypothesized, CVE significantly improved motor learning, particularly in sessions 4 and 5. This effect was reflected in a non-significant trend toward greater within-session online learning, rather than in between-session offline gains. Exploratory analyses indicate that individuals with higher cardiorespiratory fitness benefited most from CVE. Our findings highlight CVE as an effective, low-cost tool to foster motor learning in neurorehabilitation and warrant further investigation.

## Introduction

Motor skill practice is an integral component of non-pharmacological treatment for Parkinson’s disease (PD), aiming to restore and enhance skilled motor behavior and mobility^1,2^. This motor (re-)learning process requires the encoding and storage of motor information in procedural long-term memory^3^. Consequently, the efficacy of motor rehabilitation relies on long-lasting neuroplastic changes within the central nervous system^4^.

In addition to the characteristic motor symptoms (bradykinesia, rigidity, tremor, postural instability), PD also impairs motor learning^2^. Compared to age-matched neurotypical older adults, people with PD (pwPD) show deficits in both the encoding (within-session online learning) and the consolidation (between-session offline learning) of motor skills^5,6^. As cortico-striatal circuits play a crucial role in the transfer of motor information into long-term storage, some aspects of motor memory consolidation appear to be particularly affected^7^. Altered motor sequence chunking in individuals with basal ganglia stroke may further suggest that basal ganglia impairment not only affect motor but also cognitive processes involved in motor skill acquisition^8^. The underlying neuropathological mechanisms are multifactorial^5^. Experiments in humans and animal models of PD have linked motor memory deficits to reduced synaptic plasticity, including decreased long-term potentiation in the primary motor cortex^9^. In addition, altered brain activation patterns^10^ and non-motor features of PD, such as sleep disturbances, are likely to contribute^11^. Pharmacological treatments have shown limited success in addressing PD-related memory deficits and potentially even amplify the problem (dopamine overdose hypothesis)^5,12^.

Exercise has emerged as a simple, cost-effective intervention that can promote neuroplasticity and memory^13^. In young adults, accumulating evidence points at the potential of a single bout of cardiovascular exercise (CVE) to enhance motor memory formation when administered in close temporal proximity to skill practice^14^. Notably, these findings indicate that CVE has particularly beneficial effects on the consolidation of motor memories, which is the process suggested to be most significantly affected in pwPD^7^. Petzinger and colleagues^15^ postulated a model to explain potential synergistic links between CVE and goal-based motor practice to promote neuroplasticity in pwPD. Several mechanisms have been proposed by which CVE may enhance memory formation, including increased plasticity of the motor cortex^16^, upregulation of catecholamines (e.g., dopamine) as well as neurotrophic factors (e.g., brain-derived neurotrophic factor)^17^, and potentially, changes in sleep activity^18^. However, to date relatively few studies have examined the effects of CVE on the behavioral level in pwPD. Recently, we demonstrated for the first time in pwPD, that the consolidation of a newly learned balance task was enhanced by administering a single bout of CVE prior to encoding^19^. In a follow-up experiment, we were able to replicate this finding with post-encoding acute CVE^20^. Specifically, pwPD showed improved between-session offline changes after 7 days. A recent experiment extended this finding by showing that enhanced motor consolidation with CVE was paralleled by increased corticomotor excitability, a marker of neuroplasticity^21^.

Evidence on the benefits of CVE for motor memory in pwPD is scarce, and existing studies have focused exclusively on the acute effects of a single CVE session. The effects of combining skill practice with CVE over multiple practice sessions remain to be established. It could be speculated that CVE effects accumulate over repeated practice sessions. In addition, regular CVE by itself may benefit memory formation by creating an optimal environment for neuroplasticity through chronic adaptations in the central nervous system^22^. Therefore, the primary aim of this study was to examine the impact of combining motor skill practice with CVE across multiple sessions on the efficacy of motor learning in pwPD. To this end, we employed a balance learning task that has been widely used to study motor learning in ecologically valid settings^23^, has been associated with practice-induced neuroplastic changes^24,25^, has shown disease-related learning deficits^10^, and is sensitive to CVE effects in a single-session paradigm^19,20^. We hypothesized that CVE would enhance motor memory formation, resulting in improved learning of the balance task over the course of six practice sessions. The secondary aim was to test whether the potential benefits of CVE could be explained by enhanced between-session offline learning. In exploratory analyses, we examined potential effects on within-session online learning, automaticity, and self-perceived task load. We also explored associations between performance gains across practice sessions and participant characteristics (e.g., cardiorespiratory fitness) to assess their potential influence on the effects of CVE.

## Results

### Participant flow and characteristics

Forty pwPD were screened for eligibility (see Supplementary Fig. 1 for flow diagram). We excluded 13 patients after an initial telephone interview (5 did not meet inclusion criteria, 8 declined participation) and 3 patients were excluded after baseline assessment (1 patient did not meet inclusion criteria, 2 declined participation), resulting in a final sample of 24 people with mild to moderate PD enrolled for randomization. All participants completed the intervention and attended all sessions, with no adverse events during the study. Participant characteristics at baseline and follow-up assessments are presented in Table 1. In accordance with current guidelines, we refrained from significance testing for baseline differences as this not appropriate^26,27^.

**Table 1:** Participants’ characteristics; mean ± standard deviation

Outcome measures	EXE (*n* = 12)			REST (*n* = 12)			Change score comparison
	Baseline	Follow-up	Change baseline to follow-up	Baseline	Follow-up	Change baseline to follow-up	*p*
***Sociodemographic & physical characteristics***							
**Age, yrs**	61.3 ± 6.2	na	na	65.5 ± 7.1	na	na	na
**Females, n**	4	na	na	3	na	na	na
**Height, cm**	175.9 ± 6.4	na	na	173.7 ± 7.4	na	na	na
**Weight, kg**	75.7 ± 15.2	na	na	74.7 ± 11.3	na	na	na
**BMI, kg/m**^2^	24.3 ± 3.8	na	na	24.7 ± 3.3	na	na	na
***Medical information & health status***							
**Time since diagnosis, m**	59.8 ± 60.6	na	na	50.2 ± 43.3	na	na	na
**LEDD, mg**	670.9 ± 391.9	na	na	653.5 ± 366.8	na	na	na
**H&Y, pt** (higher = worse)	2.2 ± 0.4	2.1 ± 0.3	0.1 ± 0.3	2.3 ± 0.5	2.2 ± 0.4	0.1 ± 0.3	1.000
**MDS-UPDRS-III, pt** (higher = worse)	24.8 ± 10.4	23.9 ± 7.9	0.9 ± 4.3	25.5 ± 8.0	25.8 ± 6.0	−0.3 ± 4.5	0.523
***Cognitive function***							
**MoCA, pt** (higher = better)	26.5 ± 1.4	26.8 ± 2.0	0.3 ± 1.7	26.3 ± 1.6	25.3 ± 2.9	−1.0 ± 2.4	0.157
***Physical fitness and activity***							
**VO**_**2peak**_**, ml/min/kg** (higher = better)	27.2 ± 7.5	na	na	26.2 ± 5.3	na	na	na
**TMST, steps** (higher = better)	191.7 ± 30.9	192.8 ± 24.8	1.2 ± 25.7	210.7 ± 30.1	222.0 ± 51.6	11.3 ± 42.6	0.486
**FTSST, s** (higher = worse)	8.1 ± 1.9	7.5 ± 1.3	0.7 ± 1.3	7.7 ± 1.6	7.2 ± 1.1	0.5 ± 1.0	0.657
**FAB scale, pt** (higher = better)	34.5 ± 2.6	35.3 ± 2.0	0.8 ± 2.0	33.5 ± 2.6	34.3 ± 3.1	0.8 ± 1.9	0.917
**IPAQ, MET/week** (higher = more)	3550.0 ± 2900.9	2924.2 ± 2054.0	−625.9 ± 3262.1	3651.6 ± 2012.0	3669.8 ± 2110.0	18.2 ± 2215.3	0.577
***Sleep***							
**PSQI, pt** (higher = worse)	8.9 ± 2.9	7.2 ± 3.3	1.8 ± 2.8	6.9 ± 2.7	6.4 ± 3.1	0.5 ± 2.5	0.288
**ESS, pt** (higher = worse)	8.3 ± 4.0	5.7 ± 3.6	2.6 ± 3.3	9.3 ± 5.7	7.7 ± 4.9	1.7 ± 4.3	0.562

*BMI* body mass index, *LEDD* levodopa equivalent daily dose, *H&Y* Hoehn & Yahr, *MDS-UPDRS-III* MDS Unified Parkinson’s Disease Rating Scale, *MoCA* Montreal Cognitive Assessment, *VO2peak* peak oxygen consumption, *TMST* Two Minute Step Test, *FTSST* Five Times Sit to Stand Test, *FAB* Fullerton Advanced Balance Scale, *IPAQ* International Physical Activity Questionnaire, *PSQI* Pittsburgh Sleep Quality Index, *ESS* Epworth Sleepiness Scale.

### Motor learning (primary analysis)

**Fig. 1:**
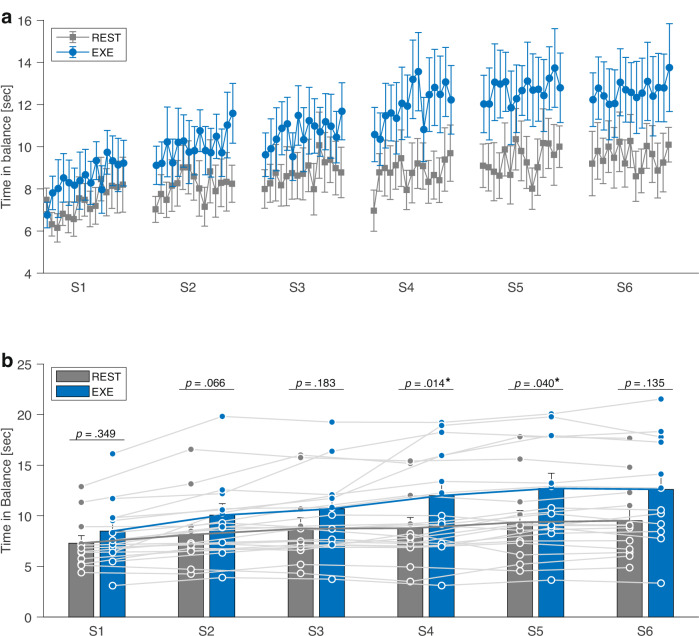
Motor performance of the balance learning task across the six practice sessions. **a** Mean time in balance for each practice trial. **b** Time in balance data averaged across each practice session (S1–S6). Both groups significantly improved their performance over the course of the 6 weeks, while the fixed effects of the linear mixed model (LMM) indicated significantly larger performance gains in sessions 4 and 5 in the cardiovascular exercise group (EXE) compared to the resting control group (REST). Error bars indicate 1 SE; *p* values represent fixed effects of the LMM; * = sig. group difference.

Our primary aim was to examine whether administering CVE immediately after motor skill practice enhances performance gains over six practice sessions. The moderate-intense CVE (EXE) group showed larger improvements from the first to the last session (4.1 ± 3.1 s; 48.9 ± 44.5%) compared to the seated rest (REST) group (2.2 ± 1.7 s; 28.9 ± 16.4%). We analyzed the data using a linear mixed model (LMM), which allowed us to model the substantial variability across individuals and sessions as random effects (Fig. 1). As expected, the best-fitting model (*mean TIB* ~ factor(group) × factor(session) + MDS-UPDRS III + (1 + session | subject) + (1 | trial) supported comparable baseline performance between groups (*p* = 0.349) and showed that participants improved their performance from week 2 (*p* < 0.05), with a higher MDS-UPDRS III score predicting poorer motor performance (*p* = 0.005) (Table 2). More importantly, CVE led to significantly larger performance gains in sessions 4 and 5 compared to rest (*p* < 0.05). A sensitivity analysis excluding one participant in the EXE group with high baseline performance confirmed the results (Supplementary Table 1 and Supplementary Fig. 2). Baseline characteristics indicated a small difference in mean age between groups and suggested a non-significant trend toward reduced performance gains across practice sessions with increasing age (*r*_*s*_(22) = −0.39, *p* = 0.059). We therefore repeated the LMM with age as an additional factor, which reproduced the beneficial effects of CVE (Supplementary Table 2). The chi-squared likelihood ratio test did not indicate an improved model fit (*p* = 0.768).

**Table 2:** Effects of the linear mixed model (LMM) testing for motor learning (*mean TIB* ~ factor(group) x factor(session) + MDS-UPDRS III + (1 + session | subject) + (1 | trial))

Random effects					
	***σ***^2^	**SD**			
**Subject (intercept)**	7.17	2.68			
**Session**	0.33	0.58			
**Trial (intercept)**	0.14	0.38			
**Residual**	4.37	2.09			

Fixed effects					
***Predictors***	***B***	***SE***_***b***_	***95% CI***	***t***	***p***
**(intercept)**	12.23	1.76	8.71, 15.73	6.95	<0.001*
**Group 1**	1.07	1.12	−1.06, 3.20	0.96	0.349
**Session 2**	0.85	0.28	0.31, 1.38	3.06	0.003*
**Session 3**	1.44	0.40	0.66, 2.22	3.60	0.001*
**Session 4**	1.48	0.55	0.41, 2.54	2.70	0.012*
**Session 5**	2.07	0.70	0.70, 3.44	2.95	0.007*
**Session 6**	2.23	0.86	0.54, 3.91	2.58	0.017*
**MDS-UPDRS III**	−0.19	0.06	−0.32, -0.07	-3.14	0.005*
**Group 1 × sessions 2**	0.73	0.39	−0.03, 1.48	1.85	0.066
**Group 1 × sessions 3**	0.77	0.57	−0.33, 1.86	1.35	0.183
**Group 1 × sessions 4**	2.03	0.77	0.53, 3.54	2.63	0.014*
**Group 1 × sessions 5**	2.15	0.99	0.21, 4.09	2.16	0.040*
**Group 1 × sessions 6**	1.89	1.22	−0.49, 4.27	1.55	0.135

group reference level = REST group; session reference level = first session; intercept = baseline performance REST group; session 2−6 = performance REST group session 2−6; group 1 = baseline performance EXE group; group 1 x session 2–6 = performance EXE group session 2−6; * = significant.

### Offline and online learning (secondary and exploratory analyses)

**Fig. 2:**
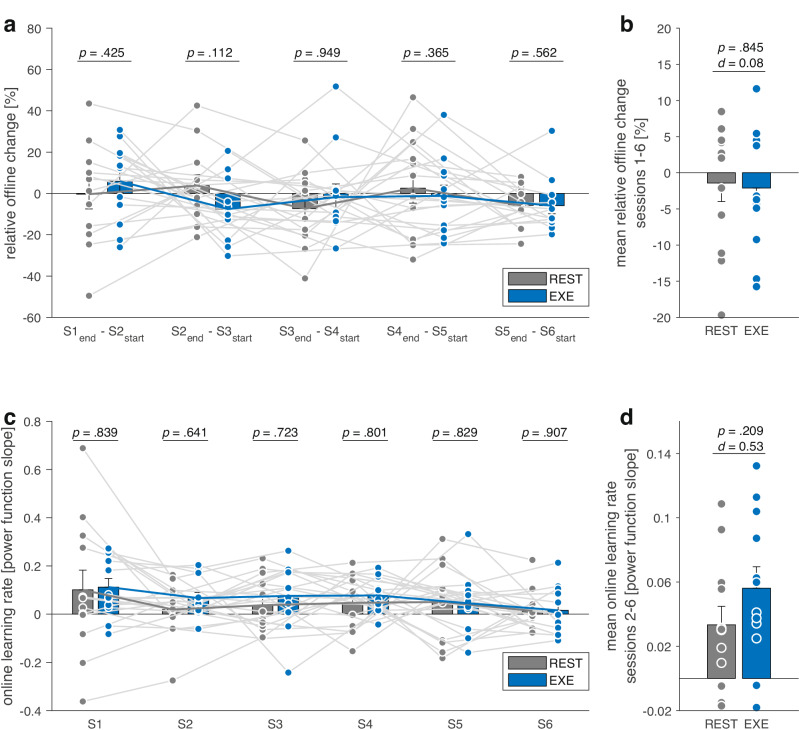
Offline and online learning. Relative between-session offline change scores (**a**) for each practice session (S1–S6) and (**b**) averaged across all sessions. The analysis did not indicate statistically significant differences in relative between-session offline change scores between the cardiovascular exercise (EXE) and rest (REST) group. Within-session online learning rates (power function slope) (**c**) for each practice session and (**d**) averaged across sessions 2–6. The EXE group descriptively showed higher within-session online learning rates averaged across sessions 2–6, although this moderate between-group effect did not reach statistical significance. Error bars indicate 1 SE; for **a**, **c** *p* values represent fixed effects of the LMM; for **b,** **d** *p* values represent results of independent samples *t*-tests.

Our secondary aim was to test whether enhanced memory consolidation explained the effects of CVE on motor learning (Fig. 2a, b). Contrary to our predictions, the LMM (*offline change score* ~ 1 + factor(group) × factor(session) + (1 | subject)) did not show any CVE effects on between-session offline change scores (Fig. 2a and Supplementary Table 3).

We then tested in an exploratory analysis if greater motor learning in the EXE group was attributed to increased performance gains during motor skill practice (Fig. 2c, d). As expected, within-session online learning rate in the first session was comparable between groups. Descriptively, participants in the EXE group exhibited higher within-session online learning rates averaged across sessions 2–6, but this moderate between-group effect was not statistically significant (*t*(22) = −1.29, *p* = 0.209, *d* = 0.53; Fig. 2d). The LMM (*online learning rate* ~ 1 + factor(group) x factor(session) + (1 | subject)) did not indicate significantly enhanced within-session online learning with CVE in any single session (Fig. 2c and Supplementary Table 5).

### Baseline characteristics and motor learning (exploratory analyses)

**Fig. 3:**
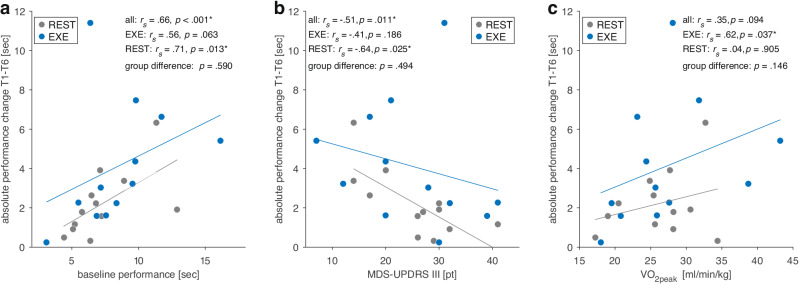
Correlations of absolute performance changes from sessions 1 to 6 for the cardiovascular exercise (EXE) and resting control group (REST). We observed greater absolute learning gains in participants with **a** higher baseline performance and **b** less severe motor symptoms (MDS-UPDRS III score). **c** In the EXE group, higher cardiorespiratory fitness (VO_2peak_) was positively associated with performance gains.

In subsequent exploratory analyses, we examined whether baseline participant characteristics influenced the effects of CVE on motor learning (Supplementary Table 7). We found that performance gains across the six sessions were significantly correlated with performance in the first session (*r*_*s*_(22) = 0.66, *p* <0.001; Fig. 3a) and MDS-UPDRS III (*r*_*s*_(22) = −0.51, *p* = 0.011; Fig. 3b), suggesting better motor learning in participants with higher baseline performance and less severe motor impairments. Notably, we also found that higher cardiorespiratory fitness was positively correlated with performance improvements in the EXE group (*r*_*s*_(10) = 0.62, *p* = 0.037; Fig. 3c). MDS-UPDRS III and VO_2peak_ were not significantly correlated (*r*_*s*_(22) = −0.28, *p* = 0.193; Supplementary Fig. 5), suggesting that both parameters represent unique aspects of functioning in relation to performance gains.

### Self-perceived task difficulty, task automaticity, and additional performance measures (exploratory analyses)

We additionally conducted an exploratory analysis of self-perceived task difficulty to assess whether exercise-induced effects on motor learning were reflected in subjective measures. On average, the EXE group visually demonstrated lower subjective task load (Supplementary Table 8 and Supplementary Fig. 6) and better subjective performance (Supplementary Table 9 and Supplementary Fig. 8) between sessions 2–6; however, this effect did not reach statistical significance. Participants who reported higher subjective performance showed significantly greater performance gains (Supplementary Fig. 7 and Supplementary Fig. 9).

To explore whether CVE led to greater automaticity in the motor task, we analyzed performance under single- and dual-task conditions in the follow-up assessment. This analysis showed that dual-task costs did not differ significantly between groups (Supplementary Fig. 10).

At follow-up assessment, we also assessed motor symptoms, global cognitive function, physical fitness, activity level, and sleep behavior to explore whether the intervention affected these parameters. Change scores from baseline revealed no significant group differences, suggesting that the CVE bout specifically enhanced motor learning (Table 1).

## Discussion

In this study, we investigated whether integrating CVE and skill practice would improve motor learning across multiple sessions in pwPD. Our findings provide preliminary evidence that motor learning improves when task practice is followed by CVE. Contrary to our expectations, this effect visually appeared to be primarily reflected in increased within-session online learning rather than in between-session offline learning, suggesting that the effect is not solely due to improved motor memory consolidation. We also found that pwPD who had higher baseline cardiorespiratory fitness experienced greater benefits from CVE. This underscores the importance of individualized approaches and the need to better understand factors that moderate the effects of CVE on motor learning.

To our knowledge, this is the first study to examine the effects of acute CVE on motor learning within a multi-session paradigm in pwPD. We hypothesized that combining motor practice with CVE within each practice session would enhance motor learning over multiple weeks of practice. While our preliminary findings must be interpreted with caution, they indeed provide support for this hypothesis. Specifically, individuals who received an additional CVE bout after practice demonstrated a steeper performance improvement, which was most evident in later sessions. To date, few studies have combined skill practice with CVE across multiple sessions. In contrast to initial results in young adults^28^, CVE did not improve motor learning in older adults^29,30^ and stroke survivors^30,31^, although some authors reported increased markers of neuroplasticity^29,31^. However, most of these studies have scheduled CVE before practice. This difference in timing complicates direct comparisons with our findings. Therefore, we demonstrate, for the first time, that CVE may be an effective and low-cost tool to enhance motor learning in typical rehabilitation settings.

Based on recent findings in pwPD, including our own^19,20^ and those of others^21^, we hypothesized that any beneficial effect of CVE, if present, would be mainly due to enhanced motor memory consolidation. However, this was not supported by our data on between-session offline changes, which partly contrasts with our previous single-session findings in pwPD^20^. Given that those studies reporting enhanced offline learning in pwPD^20,21^ and young adults^32^ have primarily observed this effect during a delayed second retention test, this discrepancy may be due to exercise-induced improvements in reconsolidation processes. Specifically, most experiments scheduled the first retention test after 24 h, followed by a delayed retention test at 7 days. CVE may lead to greater memory reactivation in the initial 24-hour retention test (e.g., through improved retrieval)^33^, potentially enhancing its effect on offline learning in the subsequent 7-day retention test^34^. Another methodological feature that differs from most studies in the field is the overlap in muscle groups used in the motor learning task and the CVE bout in our experiment. This overlap could have led to competition for neural resources, causing interference in motor-related neuronal circuits and thereby reducing the beneficial effects on consolidation processes^35^. This idea is supported by previous studies that also failed to find benefits when motor tasks and CVE engaged comparable muscle groups^36^. In addition, the lack of effects on offline changes in later sessions may indicate that measuring between-session offline changes is not sensitive enough to capture consolidation effects as practice progresses and performance approaches an asymptotic level^33^. In this line, the EXE group did indeed show a non-significant trend towards greater offline changes, but this was only the case after the first practice session. Consequently, it cannot be ruled out that CVE may have affected memory consolidation, but that this effect was masked in later session by generally dampened offline changes or asymptotic learning^33^.

Contrary to what we expected, CVE effects visually appeared to be reflected in greater within-session learning rates. While this non-significant finding needs to be interpreted with caution, it may indicate a tendency for post-encoding CVE to be linked with enhanced online processes in subsequent sessions. This is consistent with findings from Mang et al.^37^, who demonstrated improved performance gains in a retention test following a single CVE bout. The authors interpreted these results as increased re-learning or retrieval due to an exercise-induced enhancement of consolidation. A potential mechanistic explanation for this unexpected finding remains speculative. Post-encoding CVE may have modulated relevant neuronal networks, for example by increasing BDNF release, thereby creating a neurophysiological environment conducive to learning in subsequent sessions^13^. As mentioned above, these effects might also have enhanced consolidation, but they were likely more subtle in our paradigm and not detectable in between-session offline changes. Evidence from brain stimulation studies further indicates that exercise-induced neuroplasticity can improve the recall of movement strategies, thereby facilitating within-session online learning^38^. In contrast, transient effects of CVE, such as improved attention, are unlikely to account for our findings given the 7-day interval between practice sessions.

Neurophysiological research supports our notion of increased motor learning with CVE. Practice-related improvements in the stabilometer task have been linked to changes in gray and white matter, likely starting in the primary motor cortex and shifting to higher-order brain regions (e.g., prefrontal cortex)^10,24,25^. In our study, CVE may have acutely promoted these neuroplastic processes, possibly through altered cerebral blood flow^39^, while brain adaptations with longer intervention duration may have further reinforced the effects^40^, potentially explaining the larger group differences observed in later sessions. In line with this, Lehmann and colleagues^41,42^ reported enhanced learning of the same motor task in young adults following a two-week CVE intervention, attributed to increased cerebral blood flow and structural brain changes. A simple exercise-induced improvement in lower extremity function (e.g., due to overlap in muscle groups between the balance task and cycling) seems unlikely to explain enhanced motor learning, since the EXE group did not show improved performance in physical tests unrelated to motor learning (e.g., TMST, FTSST, FAB scale).

In addition, we found that individuals with higher baseline performance showed better overall motor learning, while learning was negatively affected by the severity of motor symptoms. These findings align with previous studies suggesting that motor impairments can diminish motor memory formation in pwPD^43,44^. Thus, our results highlight the importance of a minimum baseline level of physical functioning for successful learning of a complex balance skill. It could be speculated that CVE may help to reach such a level in individuals with low baseline performance, thereby facilitating subsequent motor learning.

It is plausible that ceiling effects in the capacity to acquire the motor task may have masked additional improvements in some individuals. Although the task still allowed for performance gains, some participants may already have been close to their maximal performance potential. This is supported by a decrease in online gains from session 5 onwards, suggesting asymptotic learning and probably also explaining the lack of a significant group difference in session 6. In addition, the absence of dual-task costs during follow-up testing suggests a high level of task automatization and further supports the presence of ceiling effects.

Notably, we found that individuals with higher cardiorespiratory fitness in the EXE group showed greater performance gains across the six sessions. This lends some support for the hypothesis that the effects of CVE on memory are fitness-dependent, with individuals having higher cardiorespiratory fitness experiencing greater benefits from acute CVE^45^. This may be due to greater motor cortex plasticity in response to CVE, potentially resulting from increased uptake of neurotrophic factors and brain perfusion^46^. Thus, a sufficient level of cardiorespiratory fitness may be required to benefit from acute CVE, and sedentary persons may first need to reach a critical level to fully reap these effects.

Our study has several strengths that are worth mentioning. First, we had no dropouts or adverse events throughout the study period, suggesting that the intervention is safe and feasible for pwPD with mild to moderate disease stages, provided that adequate pre-participation screening is performed. Previous studies in pwPD have focused exclusively on a single CVE session, whereas we implemented a multi-session paradigm for the first time. Unlike the majority of studies that implemented laboratory-based fine-motor skills, we used a more complex and ecologically valid task^23^. Sehm et al.^10^ demonstrated reduced motor learning in pwPD compared to age-matched neurotypical adults using the same task. This enabled us to study the effects of CVE in a task that shows disease-related learning impairments. Although our findings are preliminary, they suggest that combining skill practice with CVE may help mitigate these deficits. Therefore, CVE may offer a simpler and more cost-effective intervention to enhance motor learning compared to other currently proposed methods, such as brain stimulation.

Several limitations of the present study are important to consider when interpreting the present findings. First, the relatively small sample size of this pilot trial limits the statistical power. This may have masked potentially interesting effects, but also leave the possibility for an overestimation of some effects^47^. Additionally, motor learning data showed considerable interindividual variance (absolute improvements 0.2–11.4 s; relative improvements 5.0–178.7%). While such heterogeneity is common in neurological conditions, it can further reduce statistical power. To address this, we decided to deviate from our pre-registered analyses^48^ and conducted a more robust approach using LMMs, which better fitted the data. Nevertheless, we cannot completely rule out the possibility that individual differences in baseline performance and learning capacity may have influenced overall motor learning. Given the exploratory nature of our correlational analyses and the fact that our study was not designed to investigate the influence of participant characteristics, these findings should be treated with caution and requires replication. Finally, due to the absence of neurophysiological measures in this experiment, we can only speculate about the underlying mechanisms.

Taken together, emerging evidence suggests that acute CVE may enhance motor learning in pwPD^19–21^. Our study builds on this research by expanding these findings from single-bout paradigms to a multi-session intervention. Our results provide preliminary support for the hypothesis that motor learning improves when task practice is followed by subsequent CVE in pwPD. Although the present data need to be interpreted with caution given the limitations of this pilot study, they may have important implications for neurorehabilitation if replicated and extended. Contrary to our expectations, improved offline changes did not explain enhanced motor learning observed in the EXE group. Instead, CVE visually appeared to increase online learning, although this effect was not significant. Additionally, exploratory correlation analyses indicated that the positive effects induced by CVE may depend on the cardiorespiratory fitness level. While our findings extend the research on the effects of CVE on motor learning in pwPD, they should be interpreted cautiously and require confirmation in future, better-powered studies.

## Methods

### Participants

Twenty-four pwPD (63.4 ± 6.9 years.; 7 female; Hoehn & Yahr: 2.2 ± 0.4) were randomized in this study. Given that this was pilot work with resource constraints, a large sample was not feasible in this single-center trial. An a priori sample-size estimation based on our previous data^20^ revealed that we require *n* = 10 participants per group to detect a similar effect (*ƞ*_p_^2^ = 0.286). With an expected dropout rate of ~20%, we aimed for *n* = 24 (*n* = 12 per group).

Eligibility criteria included: (i) clinically diagnosed PD^49^; (ii) Hoehn & Yahr (H&Y) ≤ 3; (iii) MDS Unified Parkinson’s Disease Rating Scale (MDS-UPDRS) “postural stability” ≤1; (iv) naive to the motor task. Exclusion criteria were: (i) deep brain stimulation; (ii) known neurological, internal or orthopedic conditions other than PD that would interfere with the intervention; (iii) surgery of the lower extremities within the past year; (iv) Montreal Cognitive Assessment (MoCA)^50^ < 21; (v) >10 cigarettes/day, >6 cups coffee/day, and >50 g alcohol/day^51^.

Participants were recruited from the SRH Kurpfalzkrankenhaus Heidelberg, University Hospital Heidelberg, and PD Network Rhein-Neckar Plus.

### Experimental procedure

**Fig. 4:**
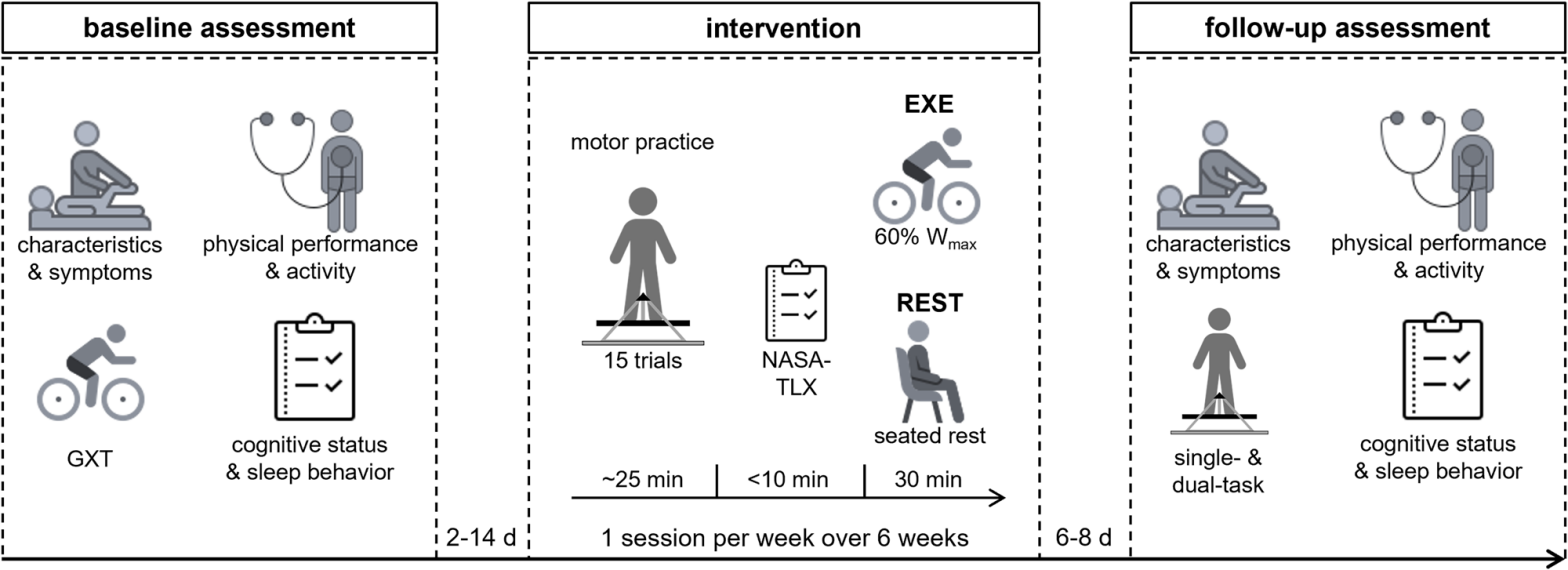
Schematic overview of the randomized controlled pilot trial. Participants attended six practice sessions over the course of six weeks. Each session consisted of practicing the motor task (stabilometer), followed by either (i) moderate-intensity cardiovascular exercise (EXE), or (ii) seated rest (REST) for 30 min, depending on randomized group assignment. Before and after the intervention participants attended a baseline and follow-up assessment, respectively; GXT = graded exercise test; *W*_max_ = maximal power output (Watts) during the graded exercise test; NASA-TLX = National Aeronautics and Space Administration-Task Load Index; icons from https://icons8.com/.

In this pre-registered randomized controlled pilot trial (see pre-registration at clinicaltrials.gov: NCT04653285), participants were randomly allocated to either (i) an experimental group (EXE) or (ii) a control group (REST), using a computer-generated block randomization (1:1 ratio) stratified by biological sex (male/female) and age (<62 years/≥62 years) (Fig. 4)^52,53^.

Participants attended six sessions of ~60 min over 6 weeks. We adapted the practice protocol as used by Sehm et al.^10^, which implemented the identical balance task and demonstrated reduced motor learning in pwPD. Each session consisted of motor task practice followed by either (i) moderate-intense CVE (EXE) or (ii) seated rest (REST) for 30 min. Baseline assessment of demographics, physical fitness and activity, motor symptoms, sleep quality, and cardiologic screening was conducted within 14 days before the intervention. Six to eight days after the last session, participants underwent a follow-up assessment.

All sessions were conducted with patients in the ON medication state, scheduled at the time of day they felt most productive, and within ±1 h each week. For daytime levodopa users, appointments were scheduled 0.5–3 h after medication intake. Participants were asked to refrain from strenuous physical activity 24 h before and after each session.

Participants were informed of the two groups but were blinded to the hypotheses. Baseline and follow-up assessments were conducted by a blinded assessor, whereas blinding of therapists during the practice sessions was not possible. All procedures were performed in accordance with the Declaration of Helsinki, and the study was approved by the ethics committee of the Medical Faculty, Heidelberg University (S-079/2022). All participants gave written informed consent prior to participation. Data were collected from September 2022 to May 2024 at the Institute of Sports and Sports Sciences, Heidelberg University.

### Baseline and follow-up assessment

Within 14 days before the first and 6–8 days after the last practice session, participants attended a baseline and follow-up assessment, respectively. The assessments included: (i) anthropometric and demographic data; (ii) motor symptoms (MDS-UPDRS III)^54^; (iii) balance (Fullerton Advanced Balance Scale)^55^; (iv) endurance (Two Minute Step Test)^56^; (v) strength (Five Times Sit to Stand Test)^57^; (vi) global cognitive status (MoCA)^50^; (vii) self-reported physical activity (International Physical Activity Questionnaire short form)^58^; (viii) daytime sleepiness (Epworth Sleepiness Scale)^59^; (ix) sleep quality (Pittsburgh Sleep Quality Index). We also assessed these measures post-intervention to explore potential changes. At follow-up assessment, we additionally evaluated dual-task performance of the motor task (see *Secondary and exploratory outcome measures*).

At a separate appointment within 2 weeks prior to the intervention, participants underwent cardiologic screening, including cardiopulmonary exercise testing in a graded exercise test. The test was performed on a cycle ergometer in an upright position. The protocol started at 25 or 50 W (pedal rate ≥60 revolutions per min) and the load was increased incrementally by 25 W every 3 min until exhaustion, while we recorded heart rate and breath-by-breath oxygen consumption to determine peak oxygen uptake (VO_2peak_).

### Motor learning task

We employed a balance learning task (stabilometer; Lafayette Instrument Europe; Loughborough; United Kingdom) to investigate motor learning in an ecologically valid and widely used experimental paradigm^23^. The task elicits characteristic learning curves, has been associated with practice-induced neuroplastic adaptations^24,25^, and reveals disease-related learning deficits in pwPD^10^. The design of the task was identical to that used in our previous work on the acute effects of CVE on motor memory in PD^19,20^. For this multi-session study, we followed the procedure described by Sehm et al.^10^, who reported reduced learning gains and altered neuroplasticity in pwPD compared to age-matched neurotypical controls. Briefly, the stabilometer is a dynamic balance task consisting of a tiltable (mediolateral) wooden platform (107 × 65 cm) with a maximum deviation of 15° to either side. We instructed participants to stand with both feet on the platform (foot position was maintained during all trials) and to keep the platform as close to the horizontal as possible during a trial (participants wore a safety harness). The trial duration was 30 s, and the platform angle was sampled at 25 Hz with an integrated electronic tilt angle measurement unit. Each session included a familiarization trial and 15 practice trials, divided into five blocks of three trials, with 60 s rest between trials and 120 s rest between blocks. We used standardized instructions, excluding any form of motivation or direction of attentional focus. Immediately after each trial, participants received feedback only on their time in balance (knowledge of result).

### Primary outcome measure

Stabilometer data were post-processed in MATLAB (R2023a; MathWorks Inc.; Natick, MA; USA). We quantified the Time in Balance (TIB), defined as the time the platform was within ±5° of horizontal during a trial^19,20^. For the evaluation of motor learning (primary outcome), we then calculated the mean TIB for each practice session (data aggregated for 15 trials), totaling six data points per participant^10^.

### Secondary and exploratory outcome measures

Based on findings from acute CVE studies, we expected that post-encoding CVE would promote offline learning in a secondary analysis. We calculated the relative change scores from the last block of each session to the first block of the subsequent session. This procedure resulted in five relative between-session offline change scores per participant (for absolute offline change scores see Supplementary Table 4 and Supplementary Fig. 3).

To further examine potential effects, we analyzed additional exploratory outcome measures. To quantify the within-session learning rate, we fitted the block data for each session separately using a general power function (MATLAB function: fit(*x*, *y*, “power1”)). The power function was defined as

1 $$y\left(x\right)=a\times {x}^{n}$$

where $n$ (power function slope) represents the within-session learning rate, $a$ (base) represents the initial session performance, and $x$ represents the practice block. This yielded six within-session online learning rates per participant (for absolute online change scores see Supplementary Table 6 and Supplementary Fig. 4).

To assess motor automaticity, participants performed an additional two blocks on the motor task at the follow-up assessment, including one familiarization trial prior to each block. The first block was performed under regular conditions, whereas the second block was performed under dual-task conditions. For the dual-task condition, participants were instructed to perform the task while simultaneously counting backwards in steps of three (starting numbers: 97, 98, and 99). Dual-task costs were calculated to explore potential effects on motor skill automaticity^60^:

2 $$dual-\,task\,\cos ts=\frac{mean\,TIB\,stabilometer\,dual-task\,block\,-\,mean\,TIB\,stabilometer\,regular\,block}{mean\,TIB\,stabilometer\,dual\,-task\,block}\times 100 \%$$

To explore differences in self-perceived task load, participants completed the National Aeronautics and Space Administration-Task Load Index (NASA-TLX) immediately after practice^61^. We analyzed the performance sub-dimension and the overall subjective task load (average of all sub-dimensions; Raw Task Load Index; RTLX), as these outcomes are correlated with balance learning^62^.

### Cardiovascular exercise and rest condition

Immediately following motor practice, participants either (i) performed moderate-intense CVE (EXE) or (ii) rested (REST) for 30 min. The CVE protocol consisted of moderate-intensity continuous upright ergometer cycling, and was identical to that used in our previous experiments, showing improved motor memory consolidation in pwPD^19,20^. After a 5-min warm-up, participants cycled for 25 min at an individually tailored intensity of 60% maximal power output (*W*_max_), while maintaining a cadence of ≥60 revolutions per min. We determined *W*_max_ as the Watts of the last fully completed stage of the GXT. We continuously measured heart rate (H10; Polar Electro; Kempele; Finland), while subjective ratings of perceived exertion (RPE; Borg scale 6–20) and blood pressure were recorded every 3 min (see Supplementary Table 10 for details of the CVE responses). To ensure moderate intensity, load was gradually adjusted if participants reported a too low (≤11) or too high (≥16) RPE. Participants in the REST group spent 30 min in a seated position while watching television.

### Statistical analysis

To investigate the effects of CVE on motor learning (primary) and offline change scores (secondary), we pre-registered two mixed analyses of variance (ANOVAs) with *group* (EXE vs. REST) as between-subject factor and *session* (mean TIB or offline change scores for sessions 1–6) as within-subject factor. We performed these analyses in a blinded manner^63^. However, inspection of the data revealed considerable interindividual variability (absolute performance gains across the six sessions ranged from 0.2 to 11.4 s; relative gains 5.0 to 178.7%). To account for this, we decided to deviate from our pre-registered analyses^48^ and re-analyze the data using linear mixed models (LMMs) (for ANOVA results see Supplementary Table 11 and Supplementary Table 12). This more robust statistical approach allowed us to model random intercepts and slopes for each participant, yielding more accurate estimates of the variance components and increasing statistical power^64^. We employed separate LMMs for the primary analysis of motor learning, the secondary analysis of offline change scores, and exploratory analyses of online learning rates and self-perceived task difficulty to examine potential exercise-induced effects. The LMMs were conducted in R (version 4.4.2) using the *lme4* package, which uses an unstructured random effects covariance structure. The dummy-coded predictors *group* (reference: REST) and *session* (reference: first session) were included in the models as fixed interaction effects^65^. We included participants as random intercepts and the group-by-session interaction was entered as random slopes (group × session | subject). For the primary analysis of motor learning, we also included MDS-UPDRS III scores as a predictor in the model, since motor symptoms correlated with learning gains (see *Results* section), and included individual trials as an additional random effect (group × session | trial). In all LMMs, we followed the recommended stepwise procedure to identify the best-fitting model, starting with the maximal model and gradually simplifying the random effect structure (removing interaction terms from the random slopes) if the model did not converge^64^. We then checked correlations of the random effects, excluding variables with high correlations close to ±1 to avoid singular fits. Finally, we used the chi-squared likelihood ratio test to check whether a more parsimonious model provided a better fit, applying a significance threshold of *p* = 0.20^66^.

In exploratory analyses, we also examined potential associations between absolute performance improvements, baseline participant characteristics, and self-perceived task difficulty. To account for the potential influence of individual participants, we performed correlation analyses using Spearman’s correlation coefficient. Lastly, we explored group differences in dual-task costs, as well as changes from baseline to follow-up assessment in motor symptoms, global cognitive function, physical fitness, activity level, and sleep behavior using separate two-tailed independent samples *t*-tests. We performed these analyses using JASP (version 0.18.1). The alpha level was set at *p* < 0.05.

## Supplementary information

Supplementary Information

## Data Availability

All data supporting the findings of this study are included in the article and its Supplementary Information. Additional data are available from the corresponding author (PW) upon reasonable request.

## Code availability

The custom-written code used to analyze the data is available from the corresponding author (PW) upon reasonable request.
