# Supplementary material for: Cardiovascular exercise enhances motor learning across multiple sessions in people with Parkinson’s disease: a randomized controlled pilot trial
Source: NPJ Sci Learn. 2025 Dec 23;11:3. doi: 10.1038/s41539-025-00391-6 (PMC12775376; doi:10.1038/s41539-025-00391-6)
Supplement: Supplementary file 1 — Supplementary Information [file 41539_2025_391_MOESM1_ESM.pdf]

Supplementary material

CARDIOVASCULAR EXERCISE ENHANCES MOTOR LEARNING ACROSS MULTIPLE SESSIONS IN PEOPLE WITH PARKINSON'S DISEASE: A RANDOMIZED CONTROLLED PILOT TRIAL

Philipp Wanner[1]*, Nicole Frisch[1], Samuel Rikus[1], Marc Roig[2,3], Simon Steib[1,4]

[1] Human Movement, Training and Active Aging Department, Institute of Sports and Sports Sciences, Heidelberg University, Heidelberg, Germany

[2] Memory and Motor Rehabilitation Laboratory (MEMORY-LAB), Feil and Oberfeld Research Centre, Jewish Rehabilitation Hospital, Montreal Centre for Interdisciplinary Research in Rehabilitation (CRIR), Laval, Quebec, Canada

[3] School of Physical and Occupational Therapy, McGill University, Montréal, Quebec, Canada

[4] Network Aging Research, Heidelberg University, Heidelberg, Germany

***Correspondence:**

Dr. Philipp Wanner

Human Movement, Training and Active Aging Department, Institute of Sports and Sports Sciences, Heidelberg University, Im Neuenheimer Feld 700, 69120 Heidelberg, Germany

Tel.: +49 (0)6221 544633

E-Mail: philipp.wanner@issw.uni-heidelberg.de

ORCID: 0000-0002-2512-921X

Supplement 1: CONSORT participant flow diagram

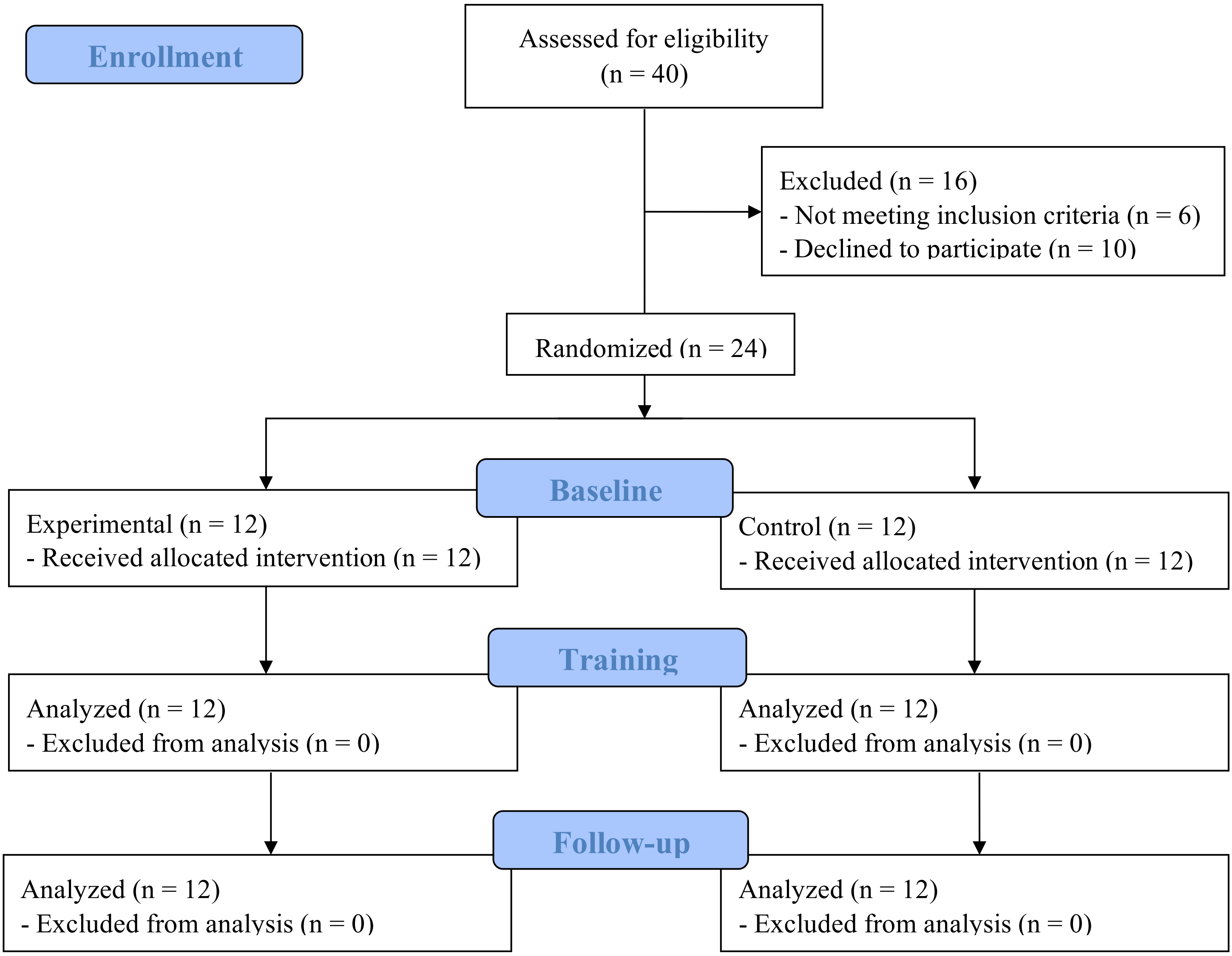

Supplementary Figure 1. CONSORT participant flow chart.

Supplement 2: linear mixed model for motor learning across the six sessions with trimmed data

Supplementary Table 1. Effects of the linear mixed model (LMM) on trimmed motor learning data, excluding one participant in the exercise group with high baseline performance (*mean TIB* ~ factor(group) x factor(session) + MDS-UPDRS III + (1 + session | subject) + (1 | trial)).

**Random effects**		
	σ^2	*SD*
**subject (intercept)**	6.05	2.46
**Session**	0.35	0.59
**trial (intercept)**	0.13	0.36
**Residual**	4.11	2.03

**Fixed effects**					
Predictors	***B***	SE_b	***95% CI***	***T***	***p***
**(intercept)**	10.38	1.73	6.87, 13.86	6.00	<.001*
**group 1**	0.62	1.05	-1.40, 2.62	0.59	.563
**session 2**	0.85	0.27	0.32, 1.38	3.09	.002*
**session 3**	1.44	0.40	0.66, 2.22	3.56	.001*
**session 4**	1.48	0.56	0.39, 2.56	2.65	.013*
**session 5**	2.07	0.72	0.67, 3.47	2.89	.008*
**session 6**	2.23	0.88	0.50, 3.95	2.52	.019*
**MDS-UPDRS III**	-0.12	0.06	-0.25, 0.00	-1.97	.063
**group 1 x sessions 2**	0.53	0.40	-0.24, 1.30	1.34	.184
**group 1 x sessions 3**	0.68	0.58	-0.45, 1.81	1.16	.252
**group 1 x sessions 4**	2.07	0.80	0.50, 3.64	2.57	.016*
**group 1 x sessions 5**	2.17	1.04	0.15, 4.20	2.10	.047*
**group 1 x sessions 6**	1.77	1.28	-0.72, 4.26	1.39	.178

group reference level = REST group; session reference level = first session; intercept = baseline performance REST group; session 2-6 = performance REST group session 2-6; group 1 = baseline performance EXE group; group 1 x session 2-6 = performance EXE group session 2-6; * = significant

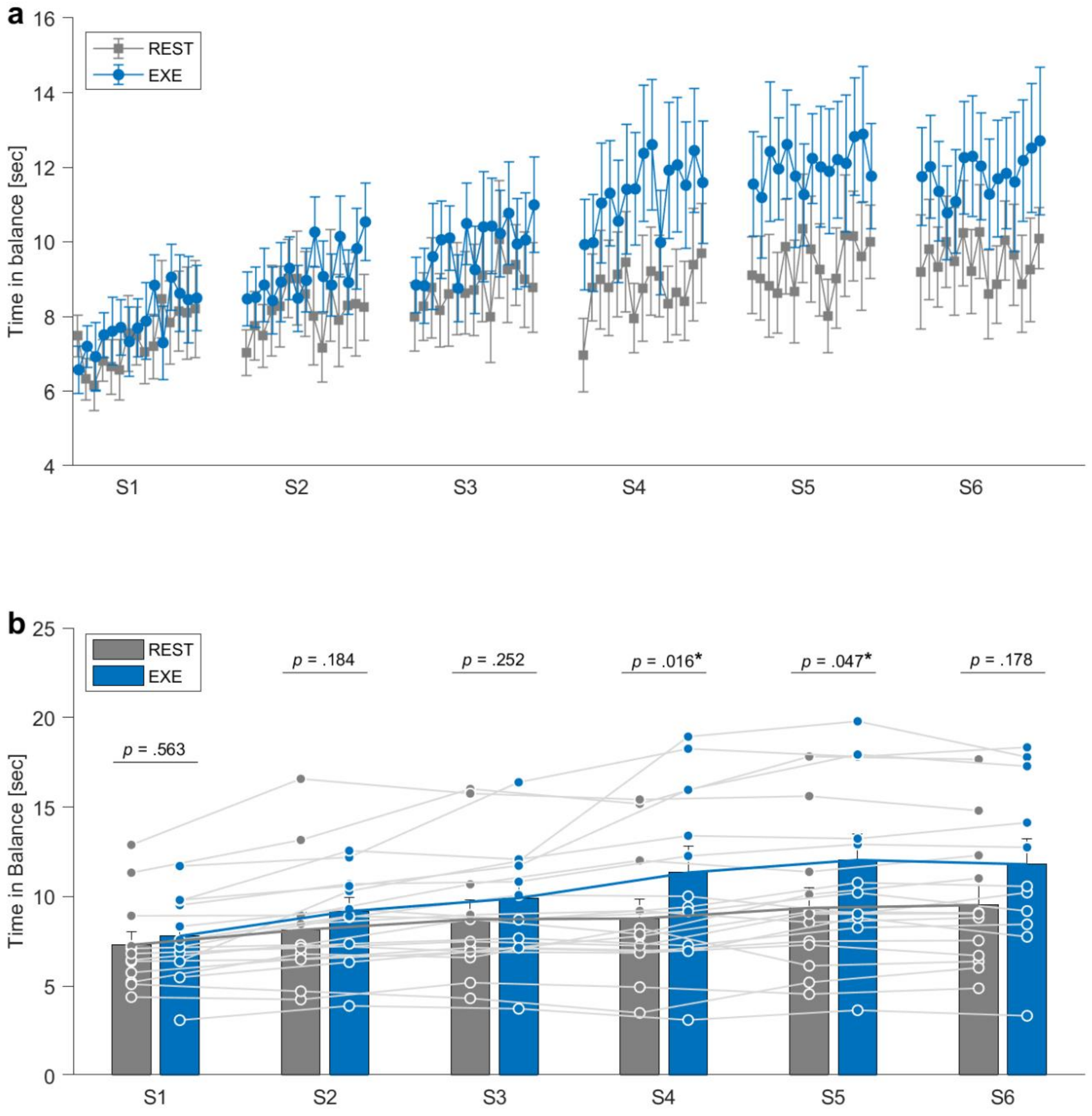

Supplementary Figure 2. Trimmed motor performance change of the balance learning task across the six practice sessions, excluding one participant in the exercise group with high baseline performance: (a) Mean time in balance for each practice trial. (b) Time in balance data averaged across each practice session (S1 – S6). Both groups improved their performance over the course of the six weeks, while the fixed effects of the LMM indicated significantly larger performance gains in sessions 4 and 5 in the exercise group (EXE) compared to the resting control group (REST). Error bars indicate 1 SE; *p*-values represent fixed effects of the LMM; * = sig. group difference.

Supplement 3: linear mixed model for motor learning across the six sessions with age as additional factor

Supplementary Table 2. Effects of the linear mixed model (LMM) testing for motor learning with MDS-UPDRS II and age as covariates (*mean TIB* ~ factor(group) x factor(session) + MDS-UPDRS III + age + (1 + session | subject) + (1 | trial)).

**Random effects**		
	σ^2	*SD*
**subject (intercept)**	7.51	2.74
**Session**	0.33	0.58
**trial (intercept)**	0.14	0.38
**Residual**	4.37	2.09

**Fixed effects**					
Predictors	***B***	***SE_b***	***95% CI***	***T***	***p***
**(intercept)**	13.72	5.82	2.87, 24.58	2.36	.029*
**group 1**	0.97	1.2	-1.28, 3.21	0.81	.430
**session 2**	0.85	0.28	0.31, 1.38	3.06	.003*
**session 3**	1.44	0.4	0.66, 2.22	3.6	.001*
**session 4**	1.48	0.55	0.41, 2.54	2.7	.012*
**session 5**	2.07	0.7	0.7, 3.44	2.95	.007*
**session 6**	2.23	0.86	0.54, 3.91	2.58	.017*
**MDS-UPDRS III**	-0.19	0.07	-0.32, -0.06	-2.81	.011*
**Age**	-0.03	0.09	-0.2, 0.15	-0.27	.789
**group 1 x sessions 2**	0.73	0.39	-0.03, 1.48	1.85	.066
**group 1 x sessions 3**	0.77	0.57	-0.33, 1.86	1.35	.183
**group 1 x sessions 4**	2.03	0.77	0.53, 3.54	2.63	.014*
**group 1 x sessions 5**	2.15	0.99	0.21, 4.09	2.16	.040*
**group 1 x sessions 6**	1.89	1.22	-0.49, 4.27	1.55	.135

group reference level = REST group; session reference level = first session; intercept = baseline performance REST group; session 2-6 = performance REST group session 2-6; group 1 = baseline performance EXE group; group 1 x session 2-6 = performance EXE group session 2-6; * = significant

Supplement 4: linear mixed model for relative offline change scores

Supplementary Table 3. Effects of the linear mixed model (LMM) on relative between-session offline change scores (*relative offline change score* ~ 1 + factor(group) x factor(session) + (1 | subject)).

**Random effects**		
	σ^2	*SD*
**subject (intercept)**	3.28	1.81
**Residual**	359.85	18.97

**Fixed effects**					
Predictors	***B***	SE_b	***95% CI***	***T***	***p***
**(intercept)**	-0.35	5.50	-10.76, 10.06	-0.06	.949
**group 1**	6.23	7.78	-8.48, 20.95	0.80	.425
**session 2-3**	4.15	7.74	-10.53, 18.83	0.54	.594
**session 3-4**	-6.98	7.74	-21.66, 7.69	-0.90	.370
**session 4-5**	2.93	7.74	-11.75, 17.61	0.38	.706
**session 5-6**	-5.42	7.74	-20.10, 9.26	-0.70	.486
**group 1 x sessions 2-3**	-17.60	10.95	-38.36, 3.16	-1.61	.112
**group 1 x sessions 3-4**	-0.71	10.95	-21.47, 20.05	-0.07	.949
**group 1 x sessions 4-5**	-9.98	10.95	-30.74, 10.78	-0.91	.365
**group 1 x sessions 5-6**	-6.38	10.95	-27.13, 14.38	-0.58	.562

group reference level = REST group; session reference level = change session 1-2; intercept = relative offline change score REST group; session = relative offline change scores REST group session 2-6; group 1 = relative offline change score EXE group; group 1 x session = relative offline change scores EXE group session 2-6

Supplement 5: linear mixed model for absolute offline change scores

Supplementary Table 4. Effects of the linear mixed model (LMM) on absolute between-session offline change scores (*absolute offline change score* ~ 1 + factor(group) x factor(session) + (1 | subject)).

**Random effects**		
	σ^2	*SD*
**subject (intercept)**	0.12	0.35
**Residual**	3.12	1.77

**Fixed effects**					
Predictors	***B***	SE_b	***95% CI***	***T***	***p***
**(intercept)**	-0.72	0.52	-1.70, 0.26	-1.39	.167
**group 1**	0.96	0.73	-0.43, 2.35	1.31	.192
**session 2-3**	0.76	0.72	-0.61, 2.13	1.05	.295
**session 3-4**	0.06	0.72	-1.31, 1.43	0.09	.933
**session 4-5**	0.54	0.72	-0.83, 1.90	0.75	.458
**session 5-6**	0.24	0.72	-1.13, 1.60	0.33	.742
**group 1 x sessions 2-3**	-1.81	1.02	-3.75, 0.12	-1.78	.078
**group 1 x sessions 3-4**	-0.54	1.02	-2.47, 1.40	-0.53	.600
**group 1 x sessions 4-5**	-1.00	1.02	-2.93, 0.93	-0.98	.330
**group 1 x sessions 5-6**	-1.40	1.02	-3.33, 0.53	-1.37	.174

group reference level = REST group; session reference level = change session 1-2; intercept = absolute offline change score REST group; session = absolute offline change scores REST group session 2-6; group 1 = absolute offline change score EXE group; group 1 x session = absolute offline change scores EXE group session 2-6

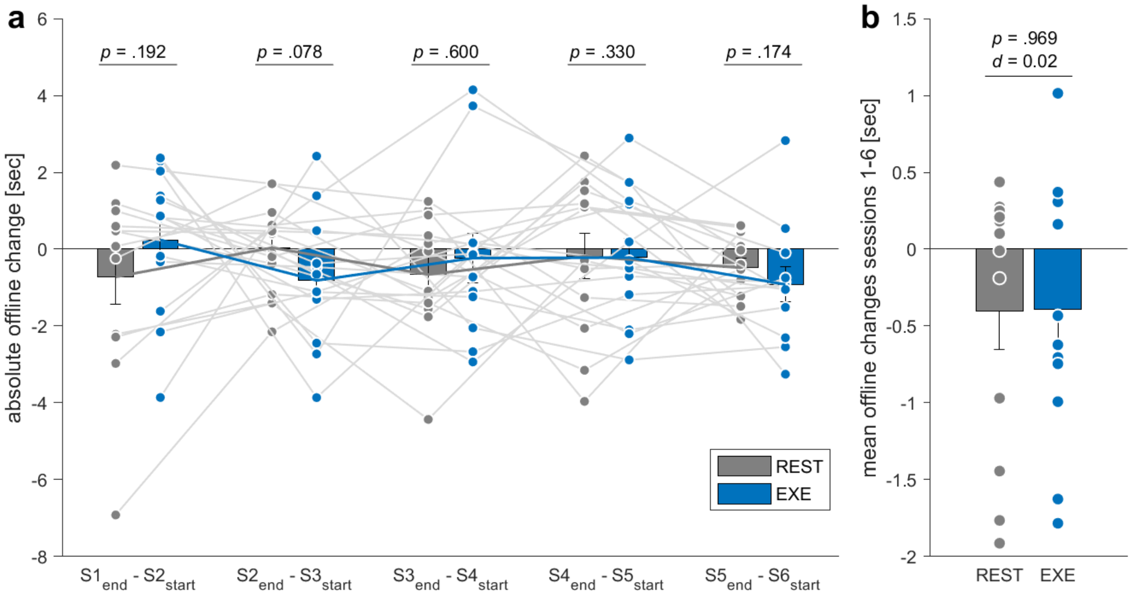

Supplementary Figure 3. Absolute between-session offline learning: Absolute between-session offline change scores (a) for each practice session (S1-S6) and (b) averaged across all sessions. Contrary to our expectations, exercise (EXE) did not lead to improved offline learning compared to resting (REST). Error bars indicate 1 SE; for Figure a *p*-values represent fixed effects of the LMM; for Figure b *p*-value represents results of independent samples *t*-test.

Supplement 6: linear mixed model for online learning rates (power function slope)

Supplementary Table 5. Effects of the linear mixed model (LMM) on within-session online learning rates (power function slope) (*online learning rate* ~ 1 + factor(group) x factor(session) + (1 | subject)).

**Random effects**		
	σ^2	*SD*
**subject (intercept)**	0.001	0.027
**Residual**	0.018	0.134

**Fixed effects**					
Predictors	***B***	SE_b	***95% CI***	***T***	***p***
**(intercept)**	0.101	0.039	0.026, 0.175	2.56	.012*
**group 1**	0.011	0.056	-0.094, 0.117	0.20	.839
**session 2**	-0.082	0.055	-0.185, 0.022	-1.50	.138
**session 3**	-0.064	0.055	-0.167, 0.039	-1.17	.244
**session 4**	-0.054	0.055	-0.157, 0.049	-0.99	.324
**session 5**	-0.050	0.055	-0.154, 0.053	-0.92	.358
**session 6**	-0.087	0.055	-0.191, 0.016	-1.60	.113
**group 1 x sessions 2**	0.036	0.077	-0.110, 0.182	0.47	.641
**group 1 x sessions 3**	0.027	0.077	-0.119, 0.173	0.36	.723
**group 1 x sessions 4**	0.020	0.077	-0.126, 0.166	0.25	.801
**group 1 x sessions 5**	-0.017	0.077	-0.163, 0.129	-0.22	.829
**group 1 x sessions 6**	-0.009	0.077	-0.155, 0.137	-0.12	.907

group reference level = REST group; session reference level = session 1; intercept = online learning rate REST group; session = online learning rate REST group session 2-6; group 1 = online learning rate EXE group; group 1 x session = online learning rate EXE group session 2-6; * = significant

Supplement 7: linear mixed model for absolute online change scores

Supplementary Table 6. Effects of the linear mixed model (LMM) on absolute within-session online change scores (*absolute online change scores* ~ 1 + factor(group) x factor(session) + (1 | subject)).

**Random effects**		
	σ^2	*SD*
**subject (intercept)**	0.65	0.81
**Residual**	3.34	1.83

**Fixed effects**					
Predictors	***B***	SE_b	***95% CI***	***T***	***p***
**(intercept)**	1.48	0.58	0.39, 2.57	2.56	.012*
**group 1**	0.23	0.82	-1.32, 1.77	0.28	.783
**session 2**	-0.61	0.75	-2.02, 0.80	-0.82	.415
**session 3**	-0.90	0.75	-2.31, 0.51	-1.20	.232
**session 4**	-0.56	0.75	-1.97, 0.85	-0.75	.456
**session 5**	-0.53	0.75	-1.95, 0.88	-0.72	.475
**session 6**	-1.51	0.75	-2.92, -0.10	-2.03	.045*
**group 1 x sessions 2**	0.21	1.05	-1.78, 2.21	0.20	.842
**group 1 x sessions 3**	0.27	1.05	-1.73, 2.26	0.26	.799
**group 1 x sessions 4**	0.65	1.05	-1.35, 2.64	0.61	.541
**group 1 x sessions 5**	-0.28	1.05	-2.28, 1.71	-0.27	.790
**group 1 x sessions 6**	0.58	1.05	-1.41, 2.58	0.56	.580

group reference level = REST group; session reference level = session 1; intercept = absolute online change score REST group; session = absolute online change score REST group session 2-6; group 1 = absolute online change score EXE group; group 1 x session = absolute online change score EXE group session 2-6; * = significant

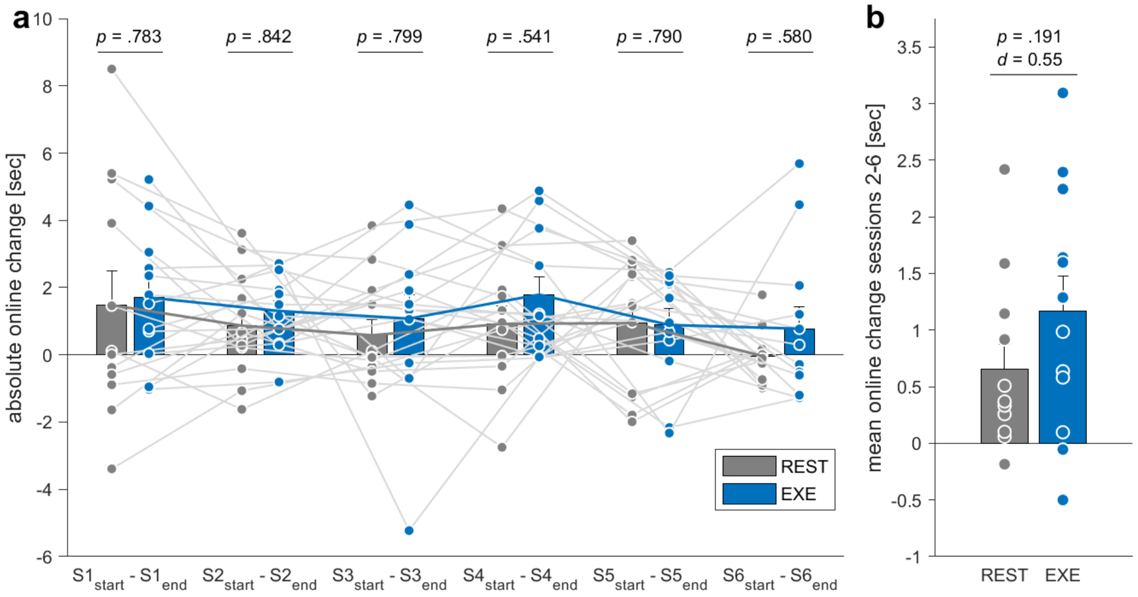

Supplementary Figure 4. Absolute within-session online learning: Absolute within-session online change scores (a) for each practice session (S1-S6) and (b) averaged across all sessions. Exercise resulted in greater average absolute within-session online learning averaged across sessions 2 and 6, but this moderate between-group effect was not statistically significant. Error bars indicate 1 SE; for Figure a *p*-values represent fixed effects of the LMM; for Figure b *p*-value represents results of independent samples *t*-test.

Supplement 8: Correlations

Supplementary Table 7. Correlations performance change scores, baseline parameters, NASA-TLX, offline, and online learning data.

		Absolute performance change from session 1 to 6			Relative performance change from session 1 to 6		
		**ALL**	**REST**	**EXE**	**ALL**	**REST**	**EXE**
**Baseline performance (performance session 1)**	r_s	.658	.706	.559	.422	.427	.315
	P	<.001*	.013*	.063	.040*	.169	.319
**MDS-UPDRS III**	r_s	-.507	-.640	-.410	-.352	-.601	-.109
	P	.011*	.025*	.186	.091	.039*	.737
**VO_{2peak}**	r_s	.350	.039	.615	.217	-.032	.406
	P	.094	.905	.037*	.307	.923	.193
**Residual VO_{2peak}, adjusted for age**	r_s	.328	-.014	.608	.210	-.035	.441
	P	.118	.974	.040*	.324	.921	.154
**Age**	r_s	-.391	-.441	-.323	-.354	-.441	-.147
	P	.059	.151	.306	.090	.151	.648
**LEDD**	r_s	-.006	.028	-.056	-.044	-.042	-.098
	P	.979	.939	.863	.840	.904	.762
**Mean self-perceived task load session 1 to 6 (NASA-TLX RTLX)**	r_s	-.174	-.270	.137	-.111	.165	.182
	P	.416	.397	.672	.607	.609	.571
**Mean self-perceived performance session 1 to 6 (NASA-TLX performance)**	r_s	-.575	-.827	-.406	-.563	-.914	-.273
	P	.003*	<.001	.193	.004*	<.001	.391
**Mean relative offline change session 1 to 6**	r_s	-.171	-.399	.112	-.039	-.231	.189
	P	.422	.201	.733	.856	.471	.558
**Mean absolute offline change session 1 to 6**	r_s	-.235	-.237	-.182	-.070	-.119	-.035
	P	.268	.391	.573	.746	.716	.921
**Mean online learning rate session 1 to 6**	r_s	.576	.559	.524	.471	.357	.517
	P	.004*	.063	.084	.021*	.256	.089
**Mean absolute online change session 1 to 6**	r_s	.715	.643	.755	.550	.469	.594
	P	<.001*	.028*	.007*	.006*	.127	.046*

ALL = all participants; REST = rest group; EXE = exercise group; r_s = Spearman's correlation coefficient; * = sig. correlation

Supplement 9: Correlation motor symptoms and cardiorespiratory fitness

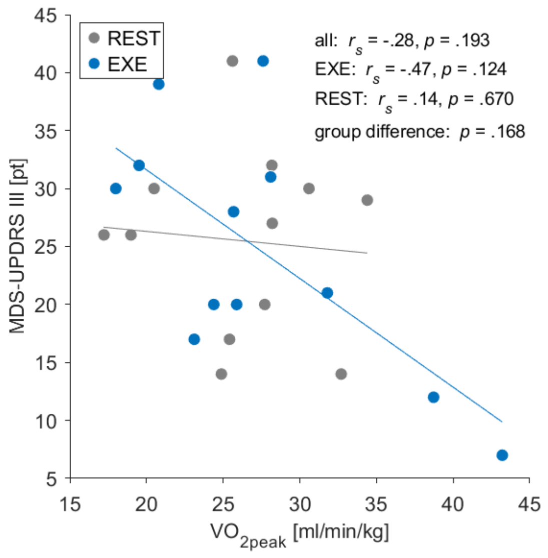

Supplementary Figure 5. Correlations between motor symptoms (MDS-UPDRS III) and cardiorespiratory fitness (VO_{2peak}) for the exercise (EXE) and resting control group (REST): More severe motor symptoms were not significantly associated with reduced cardiorespiratory fitness.

Supplement 10: linear mixed model for self-perceived task load (NASA-TLX RTLX)

Supplementary Table 8. Effects of the linear mixed model (LMM) on self-perceived task load (*NASA-TLX RTLX* ~ 1 + factor(group) x factor(session) + (1 + session | subject)).

**Random effects**		
	σ^2	*SD*
**subject (intercept)**	5.12	2.26
**Session**	0.28	0.53
**Residual**	2.43	1.56

**Fixed effects**					
Predictors	***B***	***SE_b***	***95% CI***	***T***	***p***
**(intercept)**	8.82	0.79	7.29, 10.35	11.12	<.001*
**group 1**	0.46	1.12	-1.71, 2.63	0.41	.686
**session 2**	0.46	0.65	-0.78, 1.70	0.70	.486
**session 3**	-0.35	0.71	-1.69, 0.99	-0.49	.625
**session 4**	0.58	0.79	-0.91, 2.08	0.74	.461
**session 5**	0.28	0.89	-1.42, 1.98	0.31	.756
**session 6**	0.89	1.00	-1.05, 2.83	0.89	.381
**group 1 x sessions 2**	-1.51	0.93	-3.27, 0.24	-1.63	.105
**group 1 x sessions 3**	-1.26	1.00	-3.16, 0.63	-1.26	.209
**group 1 x sessions 4**	-2.00	1.11	-4.12, 0.12	-1.80	.077
**group 1 x sessions 5**	-1.08	1.25	-3.49, 1.32	-0.87	.392
**group 1 x sessions 6**	-2.07	1.41	-4.81, 0.67	-1.47	.154

group reference level = REST group; session reference level = session 1; intercept = NASA-TLX RTLX REST group; session = NASA-TLX RTLX REST group session 2-6; group 1 = NASA-TLX RTLX EXE group; group 1 x session = NASA-TLX RTLX EXE group session 2-6; * = significant

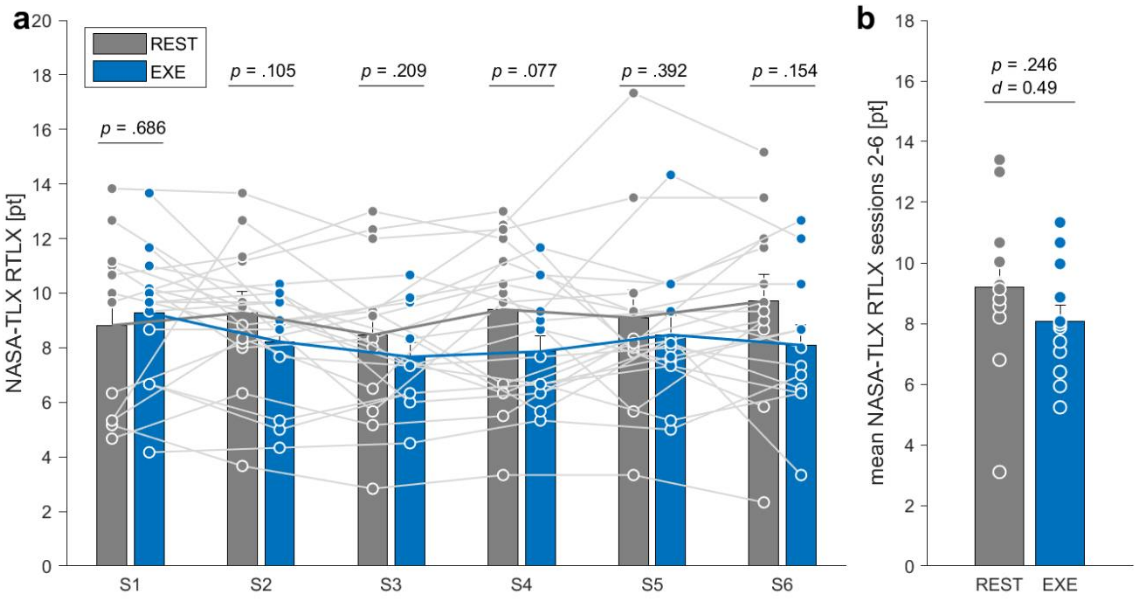

Supplementary Figure 6. Self-perceived Raw Task Load Index (RTLX), as assessed by the NASA-TLX (a) for each practice session (S1-S6) and (b) averaged across sessions 2-6. On average, participants in the exercise group (EXE) showed a lower subjective task load between sessions 2 and 6 compared to the rest group (REST), but this effect was not statistically significant. Error bars indicate 1 SE; for Figure a *p*-values represent fixed effects of the LMM; for Figure b *p*-value represents results of independent samples *t*-test.

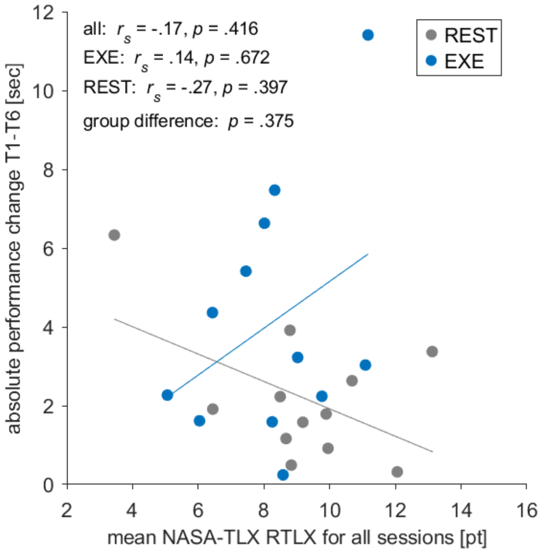

Supplementary Figure 7. Correlations between absolute performance gains from session 1-6 and self-perceived task load for the exercise (EXE) and resting control group (REST): Subjective task load, as measured by the NASA-TLX (averaged across all sessions), was not associated with greater performance gains.

Supplement 11: linear mixed model for self-perceived performance (NASA-TLX performance)

Supplementary Table 9. Effects of the linear mixed model (LMM) on self-perceived performance (*NASA-TLX performance* ~ 1 + factor(group) x factor(session) + (1 + session | subject)).

**Random effects**		
	σ^2	*SD*
**subject (intercept)**	5.75	2.40
**Session**	0.48	0.69
**Residual**	13.81	3.72

**Fixed effects**					
Predictors	***B***	SE_b	***95% CI***	***T***	***p***
**(intercept)**	11.58	1.28	9.14, 14.03	9.07	<.001*
**group 1**	-0.58	1.81	-4.04, 2.87	-0.32	0.748
**session 2**	0.08	1.53	-2.81, 2.98	0.05	0.957
**session 3**	-3.08	1.57	-6.05, -0.12	-1.97	0.052
**session 4**	-0.50	1.63	-3.59, 2.59	-0.31	0.760
**session 5**	-2.33	1.72	-5.60, 0.94	-1.36	0.179
**session 6**	-2.42	1.82	-5.92, 1.09	-1.33	0.192
**group 1 x sessions 2**	-3.25	2.16	-7.35, 0.85	-1.50	0.136
**group 1 x sessions 3**	-0.67	2.22	-4.86; 3.53	-0.30	0.764
**group 1 x sessions 4**	-1.17	2.31	-5.54; 3.20	-0.51	0.614
**group 1 x sessions 5**	-0.50	2.43	-5.12; 4.12	-0.21	0.837
**group 1 x sessions 6**	0.92	2.57	-4.04; 5.87	0.36	0.724

group reference level = REST group; session reference level = session 1; intercept = NASA-TLX performance REST group; session = NASA-TLX performance REST group session 2-6; group 1 = NASA-TLX performance EXE group; group 1 x session = NASA-TLX performance EXE group session 2-6; * = significant

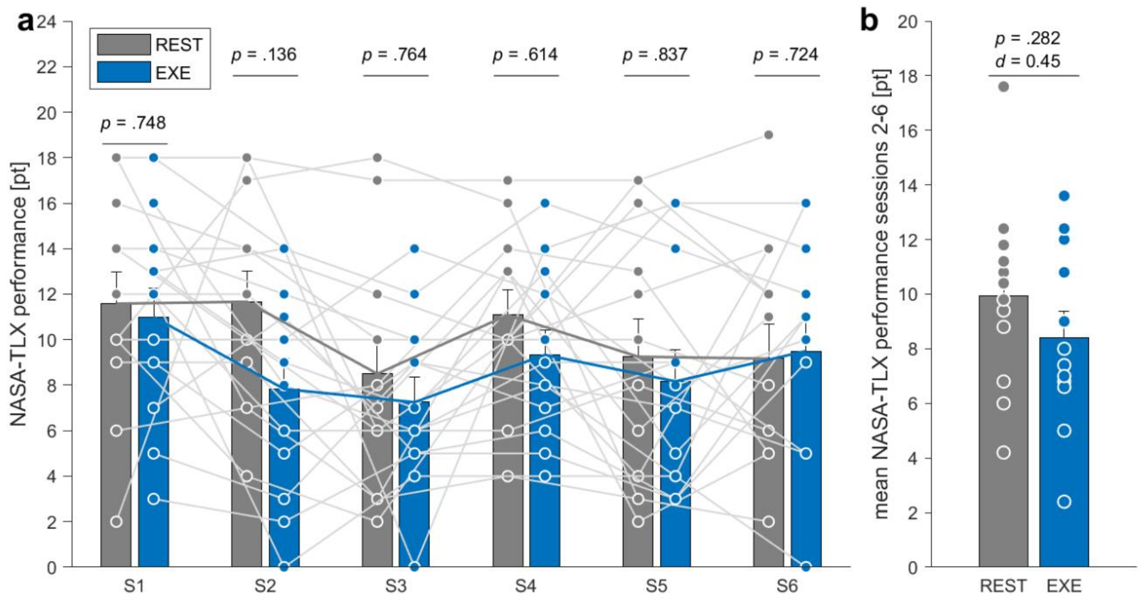

Supplementary Figure 8. Self-perceived performance, as assessed by the NASA-TLX (a) for each practice session (S1-S6) and (b) averaged across sessions 2-6. On average, participants in the exercise group (EXE) showed better subjective performance between sessions 2 and 6 compared to the rest group (REST), but this effect was not statistically significant. Error bars indicate 1 SE; for Figure a *p*-values represent fixed effects of the LMM; for Figure b *p*-value represents results of independent samples *t*-test.

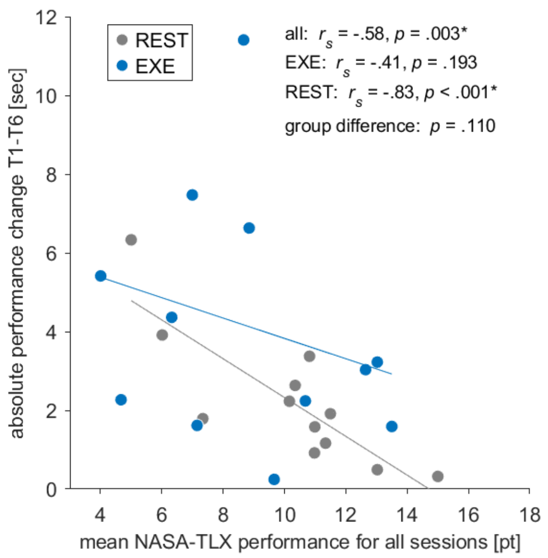

Supplementary Figure 9. Correlations between absolute performance gains from session 1-6 and self-perceived performance for the exercise (EXE) and resting control group (REST): Participants who reported better subjective performance, as measured by the NASA-TLX (averaged across all sessions), showed greater performance gains.

Supplement 12: Motor skill automaticity

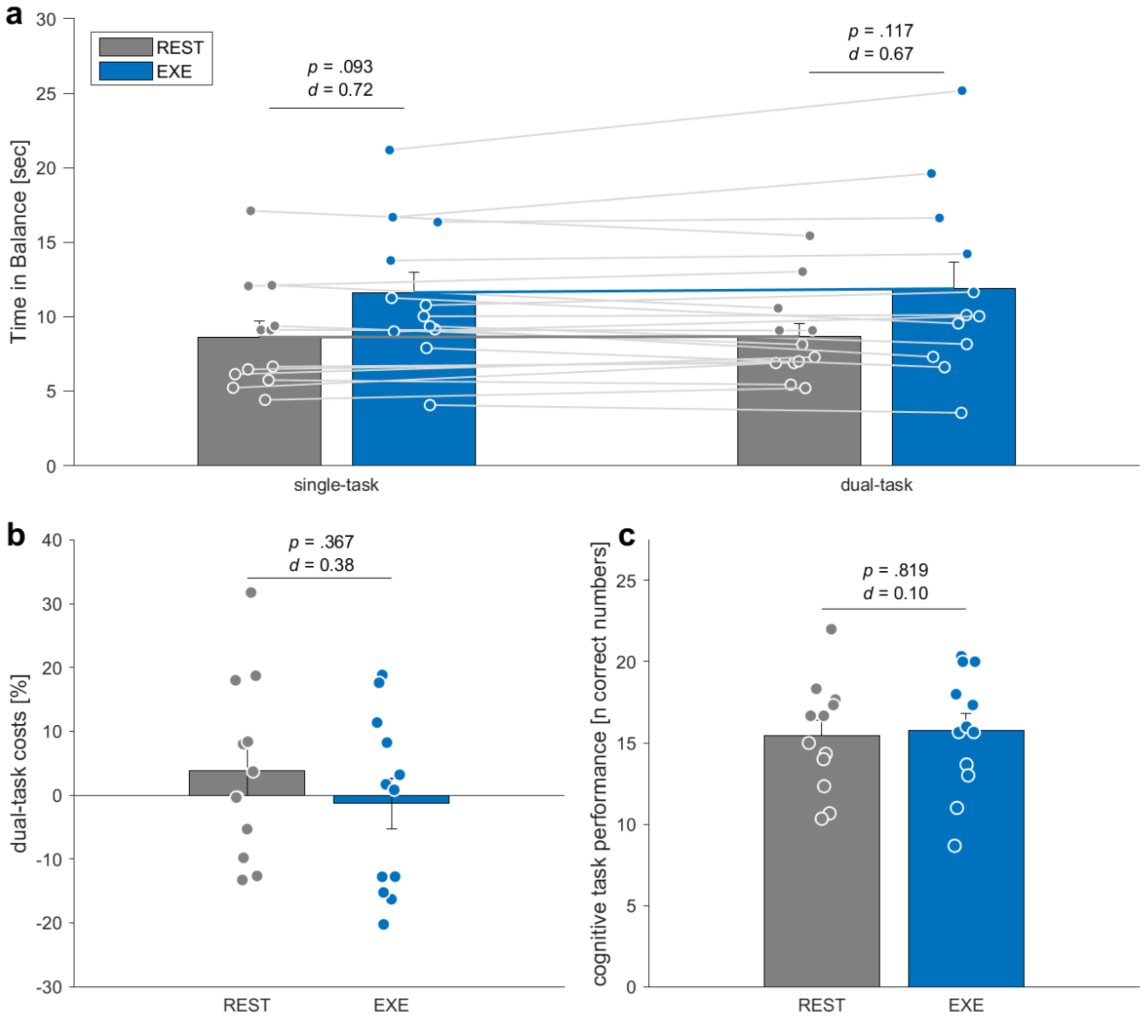

Supplementary Figure 10. Motor automaticity of the balance learning task at follow-up assessment: (a) Analyses of single- and dual-task conditions between the exercise (EXE) and resting (REST) groups revealed a non-significant moderate between-group effect favoring exercise (single-task: $t(22) = -1.755$, $p = .093$, $d = 0.72$; dual-task: $t(22) = -1.633$, $p = .117$, $d = 0.67$). However, neither group demosntarted performance decrements in the dual-task condition compared to the single-task condition. (b) Accordingly, we found no significant group differences in motor dual-task costs ($t(22) = 0.920$, $p = .367$, $d = 0.38$), (c) while performance on the cognitive task did not differ significantly ($t(22) = -0.232$, $p = .819$, $d = 0.10$). Error bars indicate 1 SE; p-values represent results of independent samples t-tests.

Supplement 13: Exercise response

Supplementary Table 10. Exercise response.

	session 1						session 2						session 3					
	heart rate (bpm)		Watt		Borg		heart rate (bpm)		Watt		Borg		heart rate (bpm)		Watt		Borg	
	warm-up	main part	warm-up	main part	warm-up	main part	warm-up	main part	warm-up	main part	warm-up	main part	warm-up	main part	warm-up	main part	warm-up	main part
**EXE**																		
Mean	91.7	111.8	47.9	77.5	10.5	14.3	92.8	112.0	45.5	78.9	9.9	14.2	91.2	110.8	48.0	80.6	9.1	13.9
SD	11.6	17.1	14.9	26.1	2.0	0.9	14.0	19.1	14.5	25.7	1.8	1.0	11.1	18.2	15.1	25.8	2.6	1.1
**REST**																		
Mean	71.3	68.7	Na	na	na	Na	72.6	71.5	na	na	na	na	71.8	72.3	na	na	na	Na
SD	13.3	12.2	Na	na	na	Na	13.3	13.0	na	na	na	na	13.8	13.0	na	na	na	Na

	session 4						session 5						session 6					
	heart rate (bpm)		Watt		Borg		heart rate (bpm)		Watt		Borg		heart rate (bpm)		Watt		Borg	
	warm-up	main part	warm-up	main part	warm-up	main part	warm-up	main part	warm-up	main part	warm-up	main part	warm-up	main part	warm-up	main part	warm-up	main part
**EXE**																		
Mean	92.7	112.1	48.0	80.8	9.0	14.1	93.5	112.8	48.0	81.6	9.4	14.2	94.1	115.1	46.6	81.3	9.1	13.9
SD	12.7	18.1	15.1	24.2	1.8	0.7	13.9	18.0	14.9	24.5	2.2	0.9	13.7	18.3	15.6	24.5	2.0	1.0
**REST**																		
Mean	70.3	68.8	Na	na	na	na	70.3	69.4	na	na	na	na	73.3	72.5	na	na	na	na
SD	12.9	12.9	Na	na	na	na	11.4	12.2	na	na	na	na	12.4	12.7	na	na	na	na

EXE = exercise group; REST = rest group; SD = standard deviation; bpm = beats per minute

Supplement 14: analysis of variance for motor learning across the six sessions

We pre-registered a mixed analysis of variance (ANOVA), with *SESSION* (*mean TIB* for each practice session T1 – T6) as within- and *GROUP* (EXE vs. REST) as between-subject factor to analyze the effects of post-encoding exercise on motor learning. Both groups significantly improved their performance over the course of the six weeks (*SESSION*: $F(1.691, 37.209) = 19.61$, $p < .001$, $\eta^2_p = .471$). The mean improvement from the first to the last session was larger in the EXE group (4.1 ± 3.1 sec; 48.9 ± 44.5 %) compared to the REST group (2.2 ± 1.7 sec; 28.9 ± 16.4 %). However, this significant *GROUP x SESSION* interaction did not survive Greenhouse-Geisser correction (*GROUP x SESSION*: $F(1.691, 37.209) = 2.53$, $p = .101$, $\eta^2_p = .103$; *Group*: $F(1, 22) = 2.35$, $p = .139$, $\eta^2_p = .097$).

Supplementary Table 11. Mixed repeated-measures ANOVA on mean time in balance across the six sessions.

Source	Sphericity correction	SS	df	MS	F	*p*	η^2_p
**Within Subjects Effect**							
SESSION	None	181.84	5.00	36.37	19.61	<.001[a]	.471
	Greenhouse-Geisser	181.84	1.69	107.51	19.61	<.001	.471
SESSION x GROUP	None	23.49	5.00	4.70	2.53	.033[a]	.103
	Greenhouse-Geisser	23.49	1.69	13.89	2.53	.101	.103
Error	None	203.97	110.00	1.85			
	Greenhouse-Geisser		37.21	5.42			
**Between Subjects Effect**							
GROUP		217.37	1	217.37	2.35	.139	.097
Error		2031.36	22	92.33			

[a] Mauchly's test of sphericity indicates that the assumption of sphericity is violated ($p < .05$)

Supplement 15: analysis of variance for relative offline change score

We pre-registered a mixed analysis of variance (ANOVA), testing for the between-subject factor *GROUP* (EXE vs. REST) and the within-subject factor *SESSION* (*offline change scores* for practice sessions 1-6) to examine whether enhanced memory consolidation could explain the effects of exercise on motor learning. The mixed ANOVA revealed no significant effect of exercise on between-session offline change scores (*GROUP*: $F(1, 22) = 0.04$, $p = .845$, $\eta^2_p = .002$; *SESSION*: $F(4, 88) = 0.85$, $p = .496$, $\eta^2_p = .037$; *GROUP x SESSION*: $F(4, 88) = 0.88$, $p = .482$, $\eta^2_p = .038$).

Supplementary Table 12. Mixed repeated-measures ANOVA on relative between-session offline change scores across the six sessions.

Source	Sphericity correction	SS	df	MS	F	p	η^2_p
**Within Subjects Effect**							
SESSION	None	1226.93	4.00	306.73	0.85	.496	.037
SESSION x GROUP	None	1260.60	4.00	315.15	0.88	.482	.038
Error	None	31666.63	88.00	359.85			
**Between Subjects Effect**							
GROUP		14.65	1	14.65	0.04	.845	.002
Error		8277.22	22	376.237			
